# LIM domain only 2 over-expression in prostate stromal cells facilitates prostate cancer progression through paracrine of Interleukin-11

**DOI:** 10.18632/oncotarget.8359

**Published:** 2016-03-25

**Authors:** Chen-Yi Jiang, Jun-Jie Yu, Yuan Ruan, Xiao-Hai Wang, Wei Zhao, Xing-Jie Wang, Yi-Ping Zhu, Yuan Gao, Kui-Yuan Hao, Lei Chen, Bang-Min Han, Shu-Jie Xia, Fu-Jun Zhao

**Affiliations:** ^1^ Department of Urology, Shanghai General Hospital, Shanghai Jiao Tong University School of Medicine, Shanghai 200080, China; ^2^ Department of Urology, Subei People's Hospital of Jiangsu Province, Clinical Medical College of Yangzhou University, Yangzhou 225001, China; ^3^ Department of Urology, Shanghai General Hospital Affiliated to Nanjing Medical University, Shanghai 200080, China

**Keywords:** prostate cancer, stromal cell, stromal-epithelial crosstalk, LIM domain only 2 (LMO2), Interleukin-11 (IL-11)

## Abstract

Mechanisms of stromal-epithelial crosstalk are essential for Prostate cancer (PCa) tumorigenesis and progression. Peripheral zone of the prostate gland possesses a stronger inclination for PCa than transition zone. We previously found a variety of genes that differently expressed among different prostate stromal cells, including LIM domain only 2 (LMO2) which highly expressed in peripheral zone derived stromal cells (PZSCs) and PCa associated fibroblasts (CAFs) compared to transition zone derived stromal cells (TZSCs). Studies on its role in tumors have highlighted LMO2 as an oncogene. Herein, we aim to study the potential mechanisms of stromal LMO2 in promoting PCa progression. The *in vitro* cells co-culture and *in vivo* cells recombination revealed that LMO2 over-expressed prostate stromal cells could promote the proliferation and invasiveness of either prostate epithelial or cancer cells. Further protein array screening confirmed that stromal LMO2 stimulated the secretion of Interleukin-11 (IL-11), which could promote proliferation and invasiveness of PCa cells via IL-11 receptor α (IL11Rα) – STAT3 signaling. Moreover, stromal LMO2 over-expression could suppress miR-204-5p which was proven to be a negative regulator of IL-11 expression. Taken together, results of our study demonstrate that prostate stromal LMO2 is capable of stimulating IL-11 secretion and by which activates IL11Rα – STAT3 signaling in PCa cells and then facilitates PCa progression. These results may make stromal LMO2 responsible for zonal characteristic of PCa and as a target for PCa microenvironment-targeted therapy.

## INTRODUCTION

Prostate cancer (PCa) is the second most frequently diagnosed cancer in men worldwide, whose incidence increases rapidly in China [1]. In early 1980s John E. McNeal proposed a zonal anatomy of the prostate by dividing the gland into peripheral zone (PZ), transition zone (TZ), central zone (CZ), and anterior fibromuscular stroma [2]. About 70% PCa originate from PZ, and the rest originate from TZ or CZ. PCa in PZ manifests stronger aggressiveness, higher pathological degree and biochemical recurrence compared to TZ [3]. However, molecular mechanisms related to the distinctive zonal characteristic of PCa remain elusive. While prostatic stromal-epithelial crosstalk is liable for this to a certain degree. The dominant cell types in prostate stroma are fibroblasts and myofibroblasts providing a homeostatic environment for epithelial/luminal cells through paracrine of essential substances including cytokines [4]. It assumes that aberrant signaling pathways mediated by cytokines in PZ may lead to PCa zonal tumorigenesis and development. Previous researches revealed that normal PZ derived stromal cells (PZSCs) as well as PCa associated fibroblasts (CAFs) possess higher tumor promotion role compared with the normal TZ derived stromal cells (TZSCs) [5–8]. Distinct functions and signaling pathways among PZSCs, TZSCs and CAFs indicate their different gene expression patterns which provide clues for further investigating the zonal characteristic of PCa.

LIM domain only 2 (LMO2) was first identified in T-cell acute lymphoblastic leukemia (T-ALL) [9, 10]. Recently, studies have indicated that LMO2 over-expression correlate with several malignancies in addition to T-ALL, including gastric cancer [11], haemangioma [12], glioma [13], pancreatic cancer [14], etc. Also, in PCa LMO2 was found positively expressed in DU145, PC-3 cells and androgen-independent CWR22 xenografts. Further, LMO2 could promote invasiveness of PCa cells through repression of E-cadherin [15]. In-depth studies using gene array move forward to confirm that LMO2 possesses a higher expression level in PZSCs and CAFs as compared to TZSCs [8, 16]. Yet, relations between stromal LMO2 and prostate tumorigenesis remain unclear.

In the present study, we attempt to elucidate potential mechanisms of tumor promotion role introduced by over-expression of LMO2 in PZSCs and CAFs. We report that stromal LMO2 over-expression can stimulate secretion of interleukin-11 (IL-11) which facilitates proliferation and invasiveness of either prostate epithelial or cancer cells via IL-11 – IL-11 receptor α (IL11Rα) – STAT3 signaling pathway. Additionally, we also suggest that impairment of miR-204-5p expression by LMO2 might be the possible reason for IL-11 up-regulation in prostate stromal cells.

## RESULTS

### LMO2 expression analyses in prostate stromal cells

**Figure 1:**
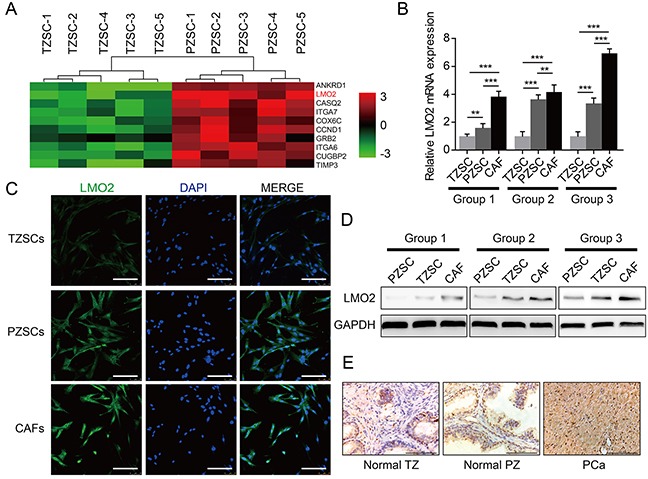
LMO2 is highly expressed in PZSCs and CAFs compared with TZSCs **A.** Different gene expression patterns between five TZSCs and five PZSCs were assessed using gene array. Heat map depicting top-10 genes that highly expressed in PZSCs compared to TZSCs and showing that LMO2 expression levels of PZSCs is higher than that of TZSCs. **B.** QRT-PCR analyses showing different LMO2 mRNA levels among TZSCs, PZSCs and CAFs derived from three cases (**P<0.01; ***P<0.001). **C.** IF analyses showing LMO2 protein localization and different expressions levels among TZSCs, PZSCs and CAFs (scale bar = 150 μm). **D.** Western blot showing different LMO2 protein levels among TZSCs, PZSCs and CAFs derived from three cases. **E.** IHC analyses showing different LMO2 protein expression in normal prostate TZ, normal prostate PZ and PCa tissues (scale bar = 100 μm).

To profile the gene expression patterns of PZSCs and TZSCs, we performed gene array analyses [8]. In all, 514 differently expressed genes were detected. Among these genes, we noticed that LMO2 is highly expressed in PZSCs compared to TZSCs (Figure 1A). Next, we measured LMO2 mRNA and protein expression using qRT-PCR (Figure 1B), Western blot (Figure 1D) and immunofluorescence (IF) (Figure 1C) for verification. Data indicated that CAFs possessed the highest expression level of LMO2, and PZSCs possessed lower LMO2 expression but still remarkably higher than TZSCs. Additionally, LMO2 protein expression in human prostate tissues was also examined using immunohistochemistry (IHC), by which the highest proportion of LMO2 positive stromal cells can be observed in PCa tissues compared with normal PZ tissues. While normal TZ tissues possess the lowest proportion of LMO2 positive stromal cells (Figure 1E, Supplementary Figure S1B). These data confirm that the expression of LMO2 has a zonal feature in prostate stroma.

### Tumor promotion role of LMO2 over-expressed prostate stromal cells

**Figure 2:**
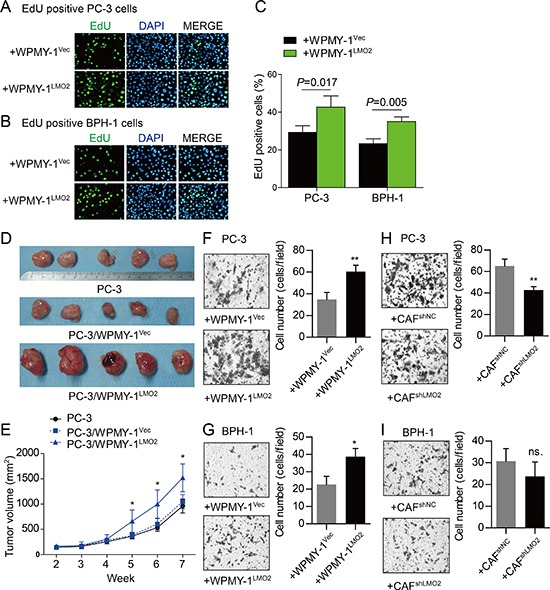
Tumor promotion role of LMO2 over-expressed stromal cells **A, B.** EdU cell proliferation assays showing proliferation change of PC-3 (A) or BPH-1 (B) cells after separately co-culturing with WPMY-1^Vec^ or WPMY-1^LMO2^ cells *in vitro*. 1×10^5^ PC-3 or BPH-1 cells were plated in the upper compartment of 6.5mm Transwell (0.4μm pore); 1×10^5^ WPMY-1^Vec^ or WPMY-1^LMO2^ cells were plated in the lower compartment. After 72h, proliferation of PC-3 or BPH-1 cells was assessed using EdU reagent. **C.** Histogram showing proliferation changes of PC-3 and BPH-1 cells after co-culture with WPMY-1^Vec^ or WPMY-1^LMO2^ cells. **D.** PC-3 homogeneous xenografts (top), PC-3/WPMY-1^Vec^ recombination xenografts (middle) and PC-3/WPMY-1^LMO2^ recombination xenografts (bottom) were excised at week seven after subcutaneous inoculation. **E.** Growth curve depicting different volumes of xenografts (*P<0.05). **F–I.** Matrigel invasion assays showing invasiveness change of PC-3 (F and H) or BPH-1 (G and I) cells after separately co-culturing with different stromal cells *in vitro*. 1×10^5^ PC-3 or BPH-1 cells were plated in the upper compartment of Matrigel coated 6.5 mm Transwell (8μm pore); 2×10^5^ stromal cells were plated in lower compartment. After co-culture for 48h, the invaded cells were stained with crystal violet.

To study functions of LMO2 in prostate stromal cells, we constructed WPMY-1^LMO2^ prostate stromal cells which over-express LMO2 (Supplementary Figure S1C). We also silenced LMO2 expression in prostate stromal cells through RNA interference. Among all three unique siRNAs we designed, siLMO2-2 possesses the highest knockdown efficiency (Supplementary Figure S1D) whose sequence was made available for CAF^shLMO2^ stable cell line construction. Afterward, we examined whether LMO2 modified stromal cells were capable of modulating the proliferation and invasiveness of prostate epithelial and cancer cells. Notably, in the *in vitro* co-culture systems EdU positive PC-3 or BPH-1 cells were increased after co-culture with WPMY-1^LMO2^ cells as compared with WPMY-1^Vec^ cells (Figure 2A–2C). CCK-8 experiments further showed that over-expression of LMO2 in WPMY-1 promoted viability of PC-3 or BPH-1 cells in co-culture system (Supplementary Figure S2A–S2B). Conversely, knockdown of LMO2 in CAFs inhibited viability of PC-3 or BPH-1 cells in co-culture system (Supplementary Figure S2C–S2D). The *in vivo* PC-3/WPMY-1 recombination xenograft model further supported the tumor promotion role of stromal LMO2 (Figure 2D). Mean volume of PC-3/WPMY-1^LMO2^ heterogeneity xenograft is larger than that of PC-3/WPMY-1^Vec^ xenograft and PC-3 homogeneous xenograft at the fifth, sixth and seventh week (Figure 2E). IHC showed that PC-3/WPMY-1^LMO2^ xenograft tissues possessed more Ki-67 positive cells than other groups (Supplementary Figure S2E–S2F). Additionally, the possible effect of stromal LMO2 on invasiveness of epithelial or cancer cells was determined using Matrigel invasion experiments. Results showed an increasing of invaded PC-3 or BPH-1 cells after co-culture with WPMY-1^LMO2^ (Figure 2F–2G). While a decreasing of invaded PC-3 cells, but not BPH-1 cells, could be observed after co-culture with CAF^shLMO2^ (Figure 2H–2I). Collectively, these data confirm a tumor promotion role of LMO2 over-expressed prostate stromal cells.

### Identification of tumor promoting cytokines stimulated by LMO2 in prostate stromal cells

**Figure 3:**
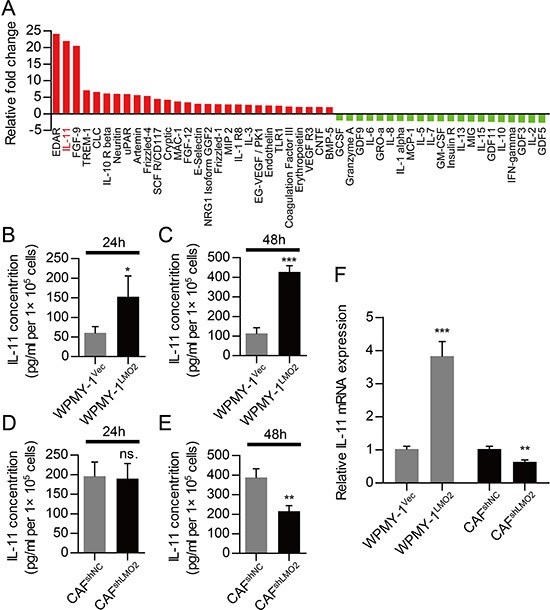
Stromal LMO2 facilitates PCa progression via paracrine of IL-11 **A.** Histogram showing increased (red columns) and decreased (green columns) proteins in supernatant of WPMY-1^LMO2^ supernatant as compared to WPMY-1^Vec^ supernatant. Proteins with more than 2-fold change are shown. **B, C.** ELISA showing different IL-11 concentration between WPMY-1^Vec^ and WPMY-1^LMO2^ supernatant after culture for 24h (B) and 48h (C). **D, E.** ELISA showing different IL-11 concentration between CAF^shNC^ and CAF^shLMO2^ supernatant after culture for 24h (D) and 48h (E). **F.** QRT-PCR analyses showing fold change of IL-11 mRNA in WPMY-1 cells after LMO2 over-expression and in CAFs after knockdown of LMO2. (ns. = not significant; * *P*<0.05; ** *P*<0.01; *** *P*<0.001).

Stromal-epithelial crosstalk in PCa may mediate by paracrine of tumor promoting cytokines. Base on the results from cells co-culture, we would like to find the possible cytokines stimulated by LMO2 in stromal cells. To this end, we performed protein array analyses, by which we identified 49 proteins with different concentrations between WPMY-1^Vec^ and WPMY-1^LMO2^ supernatant (Figure 3A, Supplementary Figure S3A). Among these proteins, the concentration of IL-11 in WPMY-1^LMO2^ supernatant up-regulated by 21.99-fold than that inWPMY-1^Vec^ supernatant. Further, ELISA assays which carried out to examine the concentration of IL-11 in supernatant of WPMY-1 cells and CAFs support the result of protein array analyses (Figure 3B–3E). Compared with control cells, WPMY-1^LMO2^ cells also possessed higher IL-11 mRNA and protein expression levels (Figure 3F, Supplementary Figure S1C). Conversely, knockdown of LMO2 in CAFs was capable of reducing IL-11 mRNA and protein expression (Figure 3F, Supplementary Figure S1D). Collectively, our data suggest that LMO2 over-expression is capable of up-regulating IL-11 mRNA expression and stimulating secretion of IL-11 in prostate stromal cells.

### Paracrine of IL-11 by prostate stromal cells facilitated PCa progression via activation of STAT3 signaling

**Figure 4:**
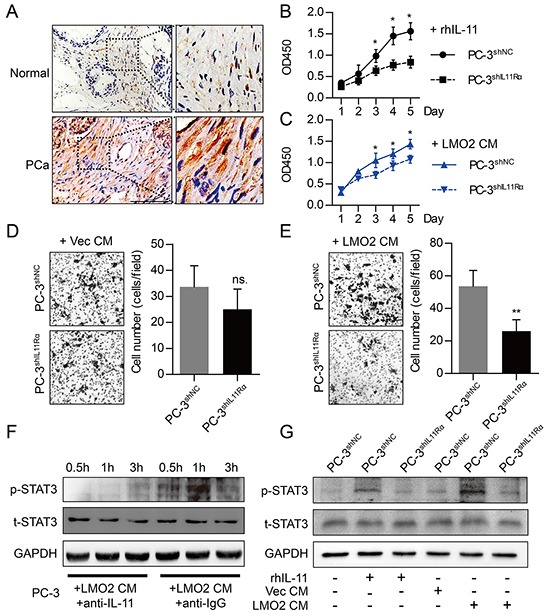
Paracrine of IL-11 by prostate stromal cells promotes proliferation and invasiveness of PCa cells via activating STAT3 signaling **A.** IHC analyses showing different IL-11 protein expression in normal prostate and PCa tissues (scale bar = 100μm). **B.** CCK-8 cell proliferation assays showing different proliferation between PC-3^shNC^ and PC-3^shIL11Rα^ cells cultured in medium with 100 ng/ml rhIL-11. **C.** CCK-8 cell proliferation assays showing different proliferation between PC-3^shNC^and PC-3^shIL11Rα^ cells cultured in conditioned medium from WPMY-1^LMO2^ cells (LMO2 CM). **D, E.** Matrigel invasion assays showing different invasiveness between PC-3^shNC^and PC-3^shIL11Rα^ cells cultured in (D) or in LMO2 CM (E). 1×10^5^ cells were plated in the upper compartment of Matrigel coated 6.5mm Transwell (8μm pore); Vec CM or LMO2 CM was added into the lower compartment. After co-culture for 48h, the invaded cells were stained with crystal violet. **F.** Western blot showing different phospho-STAT3 (p-STAT3) and total STAT3 (t-STAT3) protein levels of PC-3 cells cultured in LMO2 CM. STAT3 phosphorylation can be blocked by IL-11 neutralizing antibody (0.05 μg/ml), IgG antibody was used as control. **G.** Western blot showing different phospho-STAT3 (p-STAT3) and total STAT3 (t-STAT3) protein levels of PC-3^shNC^and PC-3^shIL11Rα^ cells cultured in different mediums. Phospho-STAT3 level increased after incubating PC-3^shNC^ with WPMY-1^LMO2^ CM or with medium containing 100ng/ml IL-11. Knockdown of shIL11Rα in PC-3 cells attenuates STAT3 protein phosphorylation. (ns. = not significant; * *P*<0.05; ** *P*<0.01).

To examine IL-11 expression in prostate tissues, we carried out IHC analyses indicating a higher IL-11 staining density in PCa tissues compared with normal prostate tissues (Figure 4A). It has been clearly discussed that biological functions of IL-11 are mediated by binding its specific receptor, IL11Rα [17]. To examine PCa promotion role of IL-11, we suppressed IL11Rα in PC-3 cells using RNA interference. CCK-8 proliferation assays indicated that either WPMY-1^LMO2^ conditioned medium (CM) and medium containing 100ng/ml recombinant human IL-11 protein (rhIL-11) was capable of promoting the proliferation of PC-3^shNC^ cells rather than PC-3^shIL11Rα^ cells (Figure 4B–4C). Furthermore, Matrigel invasion experiments suggested that invasiveness of PC-3^shNC^ cells was promoted by WPMY-1^LMO2^ CM, however, knockdown of IL11Rα in PC-3 cells reduced this function of WPMY-1^LMO2^ CM (Figure 4D–4E). Next, activation of STAT3 signaling when IL-11 exists was examined by Western blot. Notably, WPMY-1^LMO2^ CM was capable of inducing phosphorylation of STAT3 protein in PC-3 cells, while the capability could be blocked by IL-11 neutralizing antibody (Figure 4F). Moreover, knockdown of IL11Rα in PC-3 could decrease STAT3 phosphorylation mediated by rhIL-11 or WPMY-1^LMO2^ CM (Figure 4G). These data suggest that paracrine of IL-11 in stromal cells facilitates PCa progression via IL11Rα – STAT3 signaling.

### IL-11 expression in prostate stromal cells can be reversed by miR-204-5p

**Figure 5:**
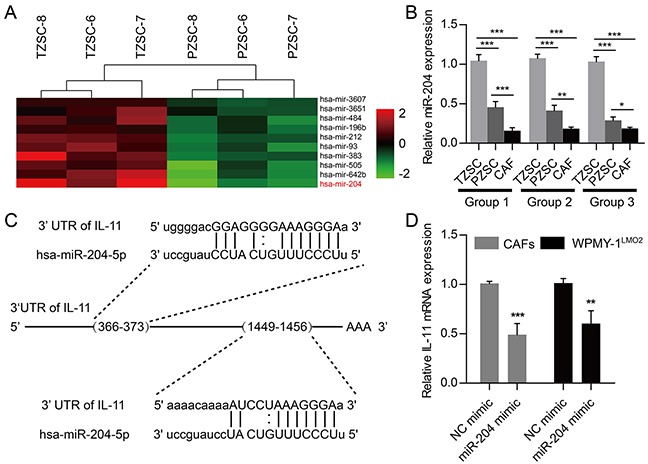
MicroRNAs profiling and suppression of IL-11 by miR-204-5p in prostate stromal cells **A.** Different miRNAs expression patterns between three TZSCs and three PZSCs were assessed using gene array. Heat map depicting top-10 genes with low expression levels in PZSCs compared to TZSCs including miR-204-5p. **B.** QRT-PCR analyses showing different miR-204-5p levels among TZSCs, PZSCs and CAFs derived from three cases. **C.** Two miR-204-5p binding sites can be found in the 3′UTR of IL-11 mRNA. **D.** QRT-PCR analyses showing fold change of IL-11 mRNA by miR-204-5p mimic transfection in CAFs and WPMY-1^LMO2^ cells. (* *P*<0.05; ** *P* <0.01; ***P <0.001).

To profile microRNAs (miRNAs) expression patterns of prostate stromal cells, we performed miRNA array analyses seeking for differently expressed miRNAs between PZSCs and TZSCs. In all, we identified 26 differently expressed miRNAs including 19 miRNAs which expressed lower levels in PZSCs than in TZSCs. MiR-204-5p was at a significantly lower expression level in PZSCs compared with TZSCs (Figure 5A). To better examine miR-204-5p expression among TZSCs, PZSCs and CAFs, we performed qRT-PCR which confirmed that expression levels of miR-204-5p in PZSCs and CAFs were less than that in TZSCs (Figure 5B). More importantly, suppression of IL-11 directly by miR-204-5p was confirmed previously, and two binding sites of miR-204-5p in the 3′UTR of IL-11 mRNA could be found (Figure 5C) [18, 19]. QRT-PCR results further demonstrated that the mRNA levels of IL-11 were decreased due to miR-204-5p mimic transfection in CAFs and WPMY-1^LMO2^ cells (Figure 5D). Collectively, data indicate that miR-204-5p can suppress IL-11 in prostate stromal cells.

### LMO2 attenuates miR-204-5p expression in prostate stromal cells

**Figure 6:**
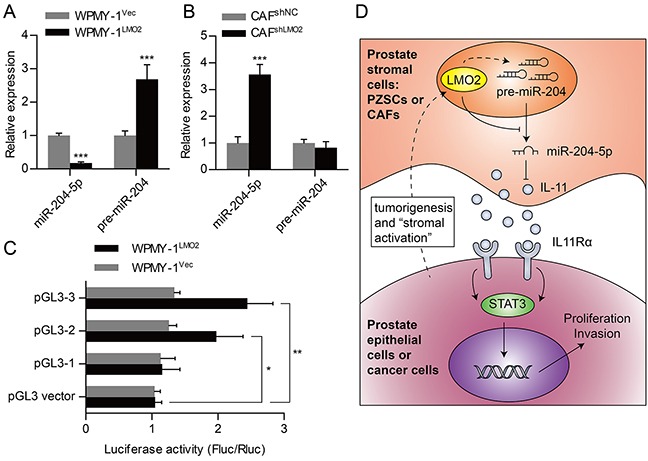
LMO2 attenuate miR-204-5p expression in prostate stromal cells **A.** QRT-PCR analyses showing fold change of miR-204-5p or pre-miR-204 expression levels between WPMY-1^Vec^ and WPMY-1^LMO2^ cells. **B.** QRT-PCR analyses showing fold change of miR-204-5p or pre-miR-204 expression levels between CAF^shNC^ and CAF^shLMO2^. **C.** Dual-luciferase assays showing different luciferase activity after co-transfection of pGL4.73 *Renilla* luciferase plasmids and different pGL3 firefly luciferase reporter plasmids into WPMY-1^Vec^ and WPMY-1^LMO2^ (* *P* < 0.05; ** *P* <0.01; ***P <0.001). **D.** Schematic diagram of PCa promotion role of LMO2 over-expressed prostate stromal cells, which shows that stromal LMO2 is capable of inducing PCa through paracrine of IL-11 and activation of STAT3 signaling pathway.

To investigate relevance between expression of miR-204-5p and LMO2 in prostate stromal cells, we performed qRT-PCR. Data showed that miR-204-5p expression was remarkably inhibited after LMO2 over-expression in WPMY-1 cells, reversely, increased due to knockdown of LMO2 in CAFs (Figure 6A–6B). LMO2 can positively or negatively modulate expression of its downstream genes. Thus we wonder whether LMO2, as a transcription factor, can regulate expression of miR-204 transcription. Interestingly, qRT-PCR data showed that LMO2 can regulated pre-miR-204 and miR-204-5p expression in an opposite way in WPMY-1 cells (Figure 6A). Next, to examine whether LMO2 could promote miR-204 gene transcription as an enhancer, we constructed gene reporter plasmids for dual-luciferase assays. Further analyses of the promoter region of human miR-204 gene implicated potential LMO2 binding sites which identified by adjacent E-box and GATA site (Supplementary Figure S3B). Then, pGL3 promoter reporter plasmids that containing different length of sequences of miR-204 gene promoter region were constructed and transfected into WPMY-1^Vec^ and WPMY-1^LMO2^ cells. Luciferase activities of pGL3-2 or pGL3-3 plasmids transfected WPMY-1^LMO2^cells were higher than that of pGL3-1 or control plasmids transfected WPMY-1^LMO2^cells (Figure 6C). Based on these results, we suggest that LMO2 could promote miR-204 gene transcription and might attenuate miR-204-5p maturation in prostate stromal cells.

## DISCUSSION

Prostate stromal cells provide a homeostatic microenvironment for normal prostate development through precise and complicated stromal-epithelial crosstalk. However, tumorigenesis promotes self-alteration of stromal cells to inclination for growth and metastasis of cancer cells [20, 21]. This process is called “stromal activation” by which an abnormal stromal-epithelial crosstalk is established [4]. Inhibition or reclamation against cancerous stromal-epithelial crosstalk offers a microenvironment-targeted strategy for PCa treatment [22]. Our previous studies have revealed that the proliferation or invasiveness of PCa cells can be induced by PZSCs and CAFs rather than TZSCs [5, 8]. Distinct gene expression patterns among TZSCs, PZSCs and CAFs implicate their different tumor promotion roles. Nevertheless, biological functions of certain genes that distinctively expressed among different stromal cells are largely unexplored. LMO2 which highly expressed in PZSCs and CAFs compared to TZSCs attracts our sight. LMO2 protein is one of the members of LIM-only gene family, which acts as a transcription factor and may perform as an oncogene [11–14]. Compared with TZSCs, PZSCs express a higher level of LMO2. So we suggest this may be accountable for the zonal characteristic of PCa. Moreover, LMO2 expression further up-regulates when PZSCs transform to CAFs after “stromal activation”, which also implicates a PCa promotion role of stromal LMO2. However, functions of LMO2 in prostate stromal cells have never been discussed. In this research, we elucidate one potential mechanism of stromal LMO2 in promoting PCa progression through stromal-epithelial crosstalk by paracrine of IL-11.

Numerous cytokines, such as transforming growth factors (TGFs) [23], fibroblast growth factors (FGFs) [24], interleukins (ILs) [25, 26], epithelial growth factor (EGF) [27], CCL2, CXCL12 [28, 29], etc., are involved in cancerous stromal-epithelial crosstalk and provide eligible microenvironment for PCa progression. Since proliferation and invasiveness of PCa cells increased after co-culture with LMO2 over-expressed stromal cells, we believe stromal LMO2 may account for PCa progression through paracrine manners. Our data based on protein array analyses suggest that stromal LMO2 can stimulate secretion of IL-11 which is a tumor promoting cytokine. IL-11, a member of the IL-6 family, can activate STAT3 signaling downstream via its unique receptor IL11Rα [17]. Previous studies confirmed serum IL-11 concentration elevation in patients with PCa [30], breast cancer [31] and lung cancer [32]. Additionally, IL-11 has the potential to promoting cancer progression in tumor microenvironment. Calon *et al.* found that secretion of IL-11 by CAFs triggers STAT3 signaling and increase metastasis in colorectal cancers [33]. Marusyk *et al.* reported that IL-11 is one of a dominant cytokines in the non-cell-autonomous driving of tumor growth [34]. Putoczki *et al.* revealed that IL-11 has a more prominent role compared to IL-6 during the progression of inflammation-associated colon and gastric cancers [35]. In PCa, Onnis *et al.* revealed that hypoxic condition can stimulate tumor cells' autocrine of IL-11 which further promote tumor progression [36]. Our study revealed the critical role of IL-11 in crosstalk between the LMO2 highly expressed stromal cells and their adjacent cancer cells. While in-depth researches necessitate understanding of the mechanisms underlying LMO2 induced IL-11 up-regulation in prostate stromal cells.

MiR-204-5p has believed to be a tumor suppressor and can attenuate IL-11 expression [18, 19]. After examining miRNAs expression of prostate stromal cells, we found that CAFs and PZSCs express lower miR-204-5p than TZSCs. We also confirmed miR-204-5p down-regulation after LMO2 over-expression in prostate stromal cells. MiR-204-5p mimic transfection was capable of suppressing IL-11 in LMO2 over-expressed stromal cells, which further suggests that LMO2 induced miR-204-5p down-regulation accounts for IL-11 accumulation. As a transcription factor, LMO2 may regulate miR-204-5p expression by transcription or post-transcription level. Previous researches in hematopoietic cells have identified several genes including *c-kit* [37], *GPA* [38], and some miRNAs [39, 40], *etc.* which could be modulated by a complex formed by LMO2, E47, SCL, LDB1, and GATA1. This complex specifically binds to adjacent E-box and GATA site in the promoter region of putative targets of LMO2 to enhance or inhibit their expression [41]. As for the interaction between LMO2 and miR-204 transcription in prostate stromal cells, gene reporter assays we performed only confirmed a promotion role of LMO2. Whether LMO2 can function directly by binding miR-204 promoter region entails more persuasive experiments such as chromatin immunoprecipitation and mutation assays. Thus, we believe stromal LMO2 over-expression may attenuate miR-204-5p expression post-transcriptionally, such as intercepting pre-miR-204 maturation or RNA-induced silencing complex (RISC) assembly.

In conclusion, the present study confirmed that LMO2 over-expression in prostate stromal cells, especially PZSCs and CAFs, is capable of suppressing miR-204-5p which entails up-regulation of IL-11. Further, increased paracrine of IL-11 by stromal cells in prostate microenvironment facilitate the proliferation and invasiveness of PCa cells via IL11Rα – STAT3 signaling (Figure 6D). These results may make stromal LMO2 responsible for zonal characteristic of PCa and as a target for PCa microenvironment-targeted therapy.

## MATERIALS AND METHODS

### Cells and human prostate specimens

PC-3 and WPMY-1 cells obtained from American Type Culture Collection (ATCC, Manassas, VA). BPH-1 cell was a gift from Prof. Ju Zhang (Nankai University, Tianjin, China). TZSCs, PZSCs and CAFs were primary cultured from normal prostate TZ tissues, normal prostate PZ tissues and PCa tissues respectively (Supplementary Figure S1A) according to procedures of primary stromal cell culture technique described previously [8, 42]. Cell lines authentication was performed according to UKCCCR Guidelines every six months, including mycoplasma detection, DNA-Fingerprinting, isozyme detection and cell vitality detection. All cells were maintained in RPMI-1640 medium (HyClone, Logan, UT) supplemented with 10% fetal bovine serum (FBS) (Gibco, Foster City, CA), 1% penicillin-streptomycin (HyClone), and cultured at 37°C in a 5% CO_2_ environment. Tissue specimens were obtained from surgery at Shanghai General Hospital between January 2011 and July 2015. The Ethical Committee of Shanghai General Hospital approved all experimental procedures, and informed consent was obtained from each patient before surgery.

### Gene array analyses

Gene array used for identifying different mRNAs expression between TZSCs and PZSCs was previously described [8]. As for identifying different miRNAs expression between TZSCs and PZSCs, we performed Affymetrix GeneChip miRNA 3.0 Array (Affymetrix, Santa Clara, CA) analyses. After Benjamini–Hochberg correction, only miRNAs under 10% false discovery rate (FDR) and more than 2-fold change difference were considered as differently expressed genes.

### SiRNAs and miRNA mimics transfection

SiRNAs, miRNA mimics and their negative controls were synthesized by GenePharma Co, Ltd. (GenePharma, Shanghai, China) and transfected into cells using Lipofectamine 2000 reagent (Life Technologies, Carlsbad, CA) according to the manufacturer's instructions. SiRNAs sequences detailed in Supplementary Table S1.

### Lentivirus construction and infection

The Coding sequence (CDS) of LMO2 mRNA (NM_005574.3) was synthesized and cloned into plenti6.3 (Life Technologies) lentivirus over-expression vectors. The siLMO2-2, siIL11Rα or negative control siRNAs were designed and cloned into pGLV (GenePharma) lentivirus shRNA vectors. After packaging, the plenti6.3-LMO2 and negative control lentivirus were used to infect WPMY-1 cells (multiplicity of infection, MOI=100) for construction of LMO2 over-expressed WPMY-1^LMO2^ and negative control WPMY-1^Vec^ cells respectively; the pGLV-shLMO2 and pGLV-shNC were used to infect CAFs (MOI=100) for construction of LMO2 suppressed CAF^shLMO2^ and negative control CAF^shNC^ cells respectively; the pGLV-shIL11Rα and pGLV-shNC were used to infect PC-3 cells (MOI=20) for construction of IL11Rα suppressed PC-3^shIL11Rα^ and negative control PC-3^shNC^ cells respectively.

### Conditioned medium (CM) preparation of prostate stromal cells

1×10^7^ prostate stromal cells were plated on 10-cm dishes in regular growth media and allowed to adhere overnight. Afterward, the supernatant was collected after culturing in serum free RPMI-1640 medium for 48h. The supernatant was centrifuged at 5,000g for 10min, filtered with 0.22mm filters (Millipore, Billerica, MA) and kept at −20°C until use. As for CM preparation, the supernatant should dilute 1:1 with fresh complete medium. 0.05 μg/ml IL-11 neutralizing antibody (500-P01, Peprotech, Rocky Hill, NJ, USA) was used to neutralize IL-11 activity in the CM. IgG antibody was used as control. Medium with 100ng/ml recombinant human IL-11 protein (rhIL-11, 218-IL, R&D, Minneapolis, MN) was used as positive control.

### Cells proliferation and invasion assays

Cell proliferation assays were performed using Cell Counting Kit-8 (CCK-8, Dojindo, Kumamoto, Japan) and EdU (RiboBio, Guangzhou, China) reagents. CCK-8 experiments were carried out for consecutive five days by adding 30 μl of CCK-8 reagent with 300μl PBS to each well, and incubated at 37°C for 2h. Viable cells were evaluated by absorbance measurements at 450nm. EdU experiments were carried out for analyzing viability of PC-3 or BPH-1 cells after co-culture with stromal cells for 72h using Cell-Light EdU Apollo 488 *In Vitro* Imaging Kit (RiboBio) according to the manufacturer's instructions. Cell invasiveness were analyzed by Matrigel (Corning) invasion experiments. 1×10^5^ PC-3 or BPH-1 cells were plated in the upper compartment of Matrigel coated 6.5mm Transwell (8μm pore) with serum free medium. After culture for 48h, the cells in the upper compartment were removed with a cotton swab, and the trans-membrane cells were fixed in 4% paraformaldehyde and stained with crystal violet. Stained cells were counted by photographing five fields/membrane at 100× magnification. All assays were performed in triplicate.

### *In vitro* cells co-culture

To construct *in vitro* cells co-culture system for proliferation and invasion assays, PC-3 or BPH-1 cells were plated in upper compartment of 6.5mm Transwell (0.4μm pore for proliferation assays, 8μm pore for invasion assays) with serum free medium, and stromal cells were plated in the lower compartment with complete medium (Supplementary Figure S1E).

### *In vivo* cells recombination

Five to six week-old male BALB/c nude mice were purchased from Animal Center of the Chinese Academy of Sciences (Shanghai, China) and maintained in a specific pathogen-free barrier facility. To construct *in vivo* cells recombination xenografts, 1×10^6^ PC-3 cells mixed with 1×10^6^ WPMY-1^Vec^ or WPMY-1^LMO2^ cells were suspended in 200μl PBS diluted Matrigel (100μl PBS+100μl Matrigel), which was subcutaneously inoculated into the flank of mice. Volumes of the xenografts were measured weekly, and the assay was terminated at seven weeks after inoculation. The animal experiments were in accordance with the US Public Health Service Policy on Humane Care and Use of Laboratory Animals, and were approved by the Scientific Investigation Board of Shanghai General Hospital.

### Quantitative real-time polymerase chain reaction (qRT-PCR)

Total RNA of prostate stromal cells was extracted using TRIzol reagent (Life Technologies). Reverse transcript PCR for mRNA was carried out using PrimeScript RT Master Mix (Takara, Otsu, Shiga, Japan) according to the manufacturer's instructions. Real-time qPCR were performed using the SYBR Premix Ex Taq (Takara). GAPDH was as an internal control for mRNAs expression. miRNA was purified using a SanPrep Column microRNA Mini-Preps Kit (Sangon Biotech, Shanghai, China) and a miRNA First Strand cDNA Synthesis Kit (Sangon Biotech) was used for Reverse transcript PCR; MicroRNAs Quantification PCR Kit (Sangon Biotech) was used for qPCR. U6 small nuclear RNA was an internal control for miRNA expression. Primer pairs detailed in Supplementary Table S1. All experiments were performed in triplicate.

### Western blot

Western blot was performed as previously described [43]. Primary antibodies against human LMO2 (AF2726, R&D), IL-11 (500-P01, Peprotech), Phospho-STAT3 (pY705) (ab76315, Abcam, Cambridge, UK), STAT3 (ab68153, Abcam) and GAPDH (5174, Cell Signaling Technology, Danvers, MA) were used in this study.

### Protein array analyses and enzyme linked immunosorbent assay (ELISA)

The secretion levels of proteins in the supernatant of WPMY-1^Vec^ and WPMY-1^LMO2^ were measured using Label-Based Human Antibody Array 507 (AAH-BLG-1, RayBiotech, Norcross, GA) according to the manufacturer's instructions. IL-11 protein levels in supernatant of prostate stromal cells were quantified using Human IL-11 ELISA kit (ELH-IL11, RayBiotech) according to the manufacturer's instructions. Cells were counted after supernatant were removed, and measurement were normalized for cell numbers. Results are given in pg/ml per 1×10^5^ cells.

### Hematoxylin and eosin (HE) staining, Immunohistochemistry (IHC) and Immunofluorescence (IF)

HE staining, IHC, and IF was performed as previously described [8, 43, 44]. Primary antibodies against human LMO2 (AF2726, R&D), IL-11 (500-P01, Peprotech) and Ki-67 (ab15580, Abcam) were used in IHC or IF analyses; FITC-conjugated anti-Goat IgG Secondary Antibody (NL997, R&D) was used for fluorescence staining. As for quantification of LMO2 positive stromal cells in IHC analyses, five ocular measuring fields within one sample were randomly chosen under a microscope at 400× magnification. The proportion of LMO2 positive stromal cells for each tissue specimen was calculated as the number of LMO2 positive stromal cells divided by the total number of stromal cells.

### Reporter plasmids construction and luciferase assays

To test whether LMO2 could induce pre-miR-204 expression via its promoter area, human pre-miR-204 promoter fragments (−953/+35, −1397/+35, and −1587/+35) were amplified by PCR from genomic DNA isolated from WPMY-1 cells. The amplified fragments were cloned into the pGL3-promoter vector (E1761, Promega, Madison, WI) at *Mlu* I and *Xho* I. Primers for vector construction detailed in Supplementary Table S1. WPMY-1^Vec^ and WPMY-1^LMO2^ cells were co-transfected with pre-miR-204 promoter vectors together with the control pGL4.73 vector (E691A, Promega). Firefly (Fluc) or *Renilla* luciferase (Rluc) activity was measured by Dual-Luciferase Reporter Assay System (E1910, Promega) 48h post-transfection according to the manufacturer's instructions. Fluc activity was standardized to the Rluc activity as an internal control. All experiments were performed in triplicate.

### Statistical analysis

All statistical analyses were carried out using SPSS (version 19.0.0, SPSS Inc., Chicago, IL). Data are shown as mean ± standard deviation. Differences between groups were analyzed using two-sided Student's *t*-test, one-way ANOVA. Statistical significance was set at *P* < 0.05.

## SUPPLEMENTARY FIGURES AND TABLE


