# Supplementary material for: LIM domain only 2 over-expression in prostate stromal cells facilitates prostate cancer progression through paracrine of Interleukin-11
Source: Oncotarget. 2016 Mar 25;7(18):26247–58. doi: 10.18632/oncotarget.8359 (PMC5041978; doi:10.18632/oncotarget.8359)
Supplement: Supplementary file 1 [file oncotarget-07-26247-s001.pdf]

SUPPLEMENTARY FIGURES AND TABLE

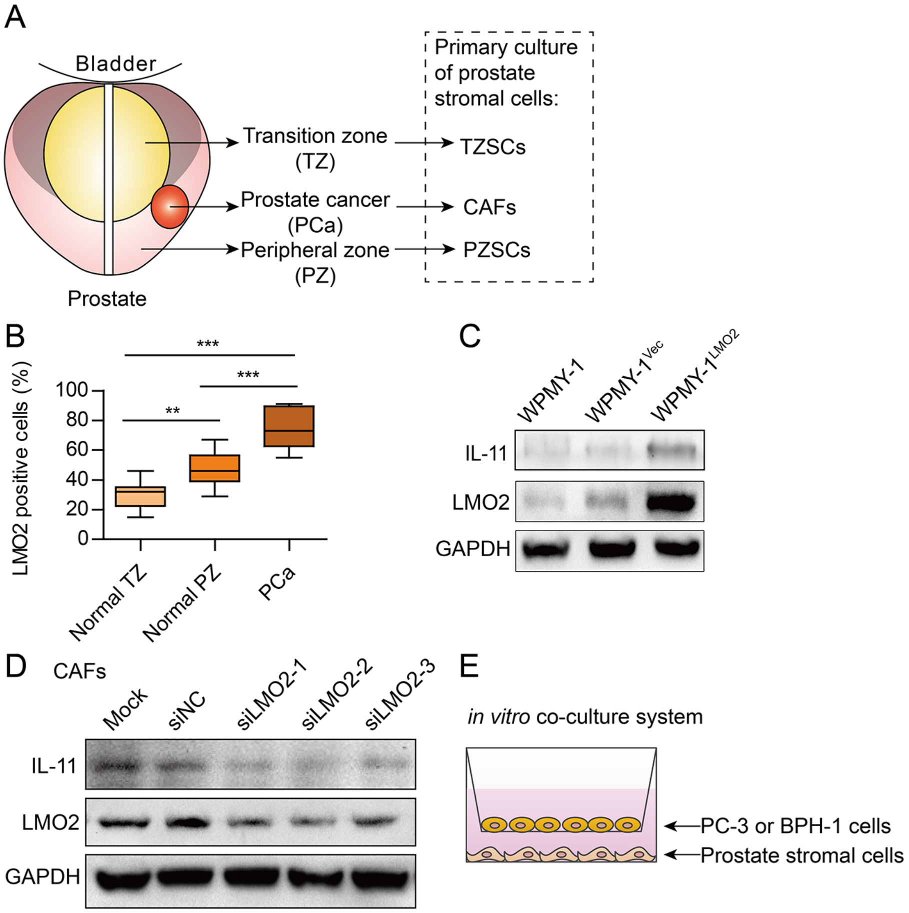

Supplementary Figure S1: A. Procedures of prostate stromal cells primary culture on the basis of zonal anatomy of a prostate gland. PZSCs and TZSCs were primary cultured from prostate peripheral zone (PZ) and transition zone (TZ) tissue respectively. CAFs were primary cultured from PCa tissue. **B.** Box plot showing different proportions of LMO2 positive stromal cells in normal prostate TZ, normal prostate PZ and PCa tissues assessed by IHC (** $P < 0.01$; ***$P < 0.001$). **C.** Western blot showing LMO2 and IL-11 protein expression levels in WPMY-1, WPMY-1^{Vec} and WPMY-1^{LMO2} cells. WPMY-1 and WPMY-1^{Vec} cells express nearly undetectable level of LMO2 protein, while WPMY-1^{LMO2} express elevated level of LMO2 protein. LMO2 over-expression in WPMY-1^{LMO2} further up-regulates intracellular IL-11 protein expression. **D.** Western blot showing LMO2 and IL-11 protein expression change after LMO2 siRNAs transfection. Three different siRNA sequences were used and siLMO2-2 had the highest knockdown efficiency. **E.** Schematic diagram showing construction of *in vitro* co-culture system using 0.4μm pore Transwell. BPH-1 or PC-3 cells were plated in upper compartment share in the same medium environment with prostate stromal cells plated in lower compartment.

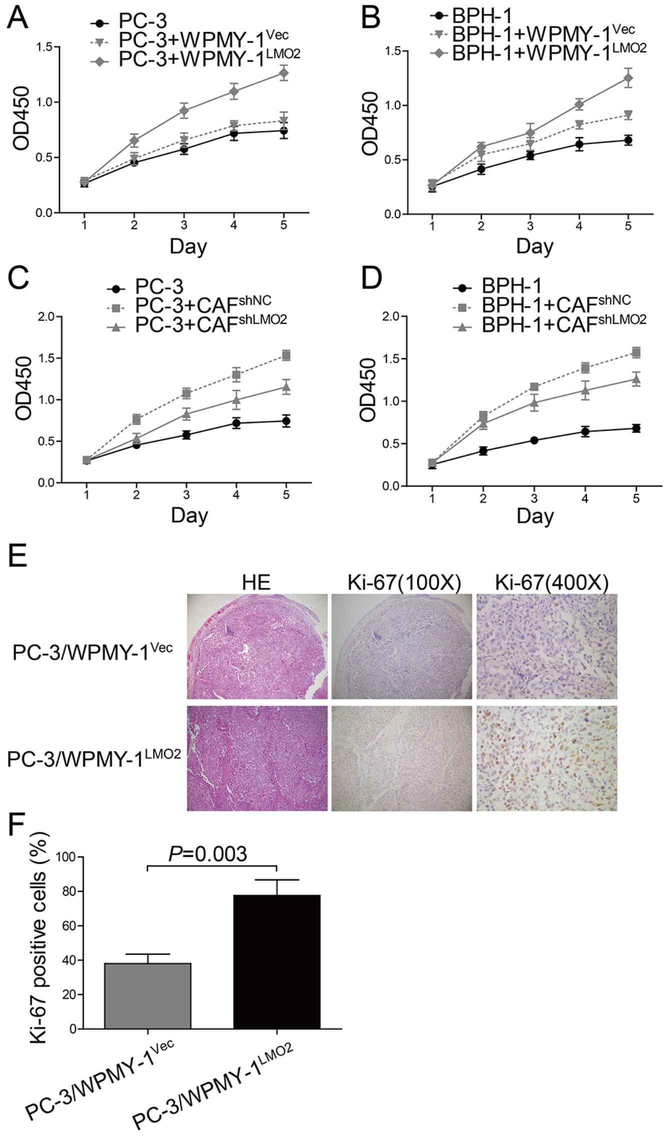

Supplementary Figure S2: A–D. CCK-8 cell proliferation assays showing different proliferation of PC-3 or BPH-1 cells when cultured alone or with different prostate stromal cells. 5×10^3 cells/well PC-3 or BPH-1 cells were plated in the upper compartment of 6.5mm Transwell (0.4µm pore) and 5×10^4 cells/well stromal cells were plated in the lower compartment. CCK-8 assays were carried out for consecutive five days. **E.** HE staining of PC-3/WPMY-1^{Vec} recombination xenografts and PC-3/WPMY-1^{LMO2} recombination xenografts and IHC analyses showing Ki-67 expression in these tissues. **F.** Compared with PC-3/WPMY-1^{Vec} xenografts, the proportion of Ki-67 positive cells is higher in PC-3/WPMY-1^{LMO2} xenografts.

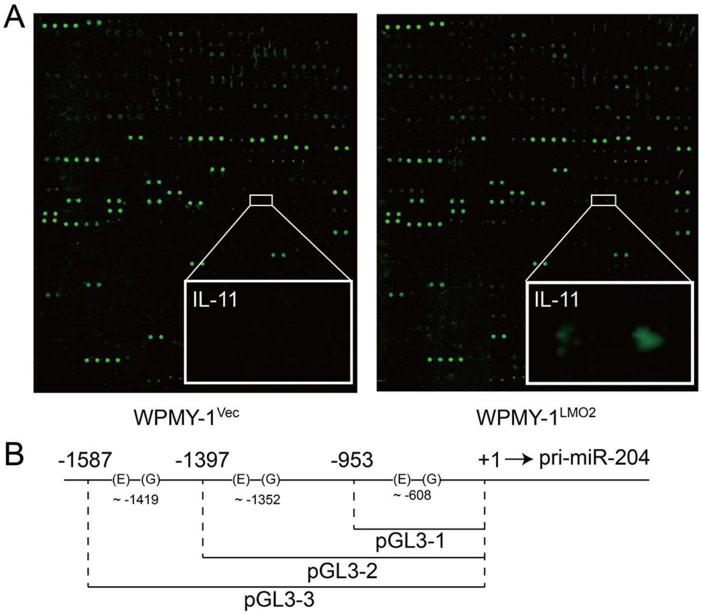

Supplementary Figure S3: A. Scanning image of protein array slides. IL-11 signals were magnified and shown in the white box. **B.** Schematic diagram showing the promoter region of miR-204 gene (chromosome 9: 70812084 - 70810085) containing adjacent E-box (E) and GATA site (G) which could be the potential binding site for LMO2 protein, and sequences used for luciferase reporter plasmids construction.

Supplementary Table S1: Primers and siRNAs

Applications	Gene	Direction	5′-3′
qRT-PCR	LMO2	forward	CTGAAGGCCATCGACCAGTA
primers		reverse	TTGTCACAGGATGCGCAGAG
	IL-11	forward	CACCATAGCAAACTGGAAACAA
		reverse	TCCTTAGCCTCCCTGAATGAC
	Pre-miR-204	forward	GTGACTCGTGGACTTCCCTTT
		reverse	ATTGAACGTCCCTTTGCCTTC
	GAPDH	forward	GAAGGTCGGAGTCAACGGATT
		reverse	CGCTCCTGGAAGATGGTGAT
	miR-204-5p	forward	AGGCGGTTCCCTTTGTCATCCT
		reverse	GTGCAGGGTCCGAGGT
	U6	forward	CTCGCTTCGGCAGCACA
		reverse	AACGCTTCACGAATTTGCGT
siRNA	siLMO2-1	sense	UGUAUCACCUGGAAUGUUU
		antisense	AAACAUUCCAGGUGAUACA
	siLMO2-2	sense	ACGAGUGGACUAAGAUCAA
		antisense	UUGAUCUUAGUCCACUCG
	siLMO2-3	sense	GGACUAAGAUCAAUGGGAU
		antisense	AUCCCAUUGAUCUUAGUCC
	siIL11Rα	sense	UGGAGCCAGUACCGGAUUAAU
		antisense	ACCUCGGUCAUGGCCUAAUUA
	siNC	sense	UUCUUCGAACGUGUCACGUTT
		antisense	ACGUGACACGUUCGGAGAATT
Gene clone	-953/+35	forward	GACATTCAACCACATCAGAA
		reverse	GAAGTCCACGAGTCACAT
	-1397/+35	forward	AATGATACTTCCTCCAGCAA
		reverse	GAAGTCCACGAGTCACAT
	-1587/+35	forward	TCAATTCCTATCGTCTCCTG
		reverse	GAAGTCCACGAGTCACAT
